# Exploration of extracorporeal shock wave therapy in adjunctive therapy for temporomandibular disorder

**DOI:** 10.1097/JS9.0000000000001967

**Published:** 2024-07-17

**Authors:** Kegui Hou, Hao Wang, Wenpeng Song

**Affiliations:** a Beijing Shunyi District Hospital; b Department of Stomatology, Beijing Tiantan Hospital, Capital Medical University, Beijing, People’s Republic of China

*Dear Editor,*

We were interested to read the study by Tang *et al*.^1^ entitled ‘The efficacy of extracorporeal shock wave therapy for knee osteoarthritis: an umbrella review’. This study found that extracorporeal shock wave therapy (ESWT) can effectively improve pain and function in patients with knee osteoarthritis through a comprehensive evaluation of eight meta-analyses. The mechanism of this efficacy may be related to the various biological effects of ESWT, including enhancing local microcirculation and oxygen supply, promoting the release of anti-inflammatory cytokines, inhibiting fibrosis and dissolving calcium deposits, and promoting the secretion of synovial fluid by chondrocytes and synoviocytes, thereby reducing friction between bone and cartilage^1^.

Temporomandibular joint disorder (TMD) is a group of disorders affecting the temporomandibular joint, masticatory muscles, and surrounding structures. Similar to knee osteoarthritis, TMD manifests as pain and functional limitations (including limited mouth opening, abnormal mouth opening pathways, and joint sounds), negatively impacting the quality of life of patients. Although several clinical practice guidelines have emerged in recent years to support the management and treatment decisions for TMD, their recommendations are not consistent^2^. Treating TMD remains challenging. Currently, there is still a lack of a single, ideal disease management approach.

The pathogenesis of TMD is complex and not yet fully understood. In recent years, some clues have gradually revealed partial similarities in the pathogenesis between temporomandibular joint osteoarthritis (TMJOA) and knee osteoarthritis. For example, pathological degenerative remodeling, including changes in the shape and overall size of joint components, can also be observed in TMJOA. Upregulation of pro-inflammatory factors such as TNF-α, IL-1, and IL-6, along with a decrease in anti-inflammatory capacity, abnormalities in certain nociceptive neuropeptides (such as substance P, CGRP, neuropeptide Y, vasoactive intestinal peptide), and oxidative stress damage are also involved in the occurrence, development, and manifestation of pain symptoms in TMD. Additionally, several types of cell death mechanisms commonly seen in the pathogenesis of knee osteoarthritis, such as apoptosis, ferroptosis, and necroptosis, have also been found in cells in the joint area of TMJOA.

Based on the effectiveness of ESWT in the treatment of knee joint diseases and the similarity between knee osteoarthritis and TMD, ESWT is expected to be applied in the clinical practice of TMD treatment. Currently, a small number of studies have focused on the value of ESWT in TMD treatment. In 2019, Kim *et al*.^3^ conducted a study based on a TMJOA rat model and found that ESWT could protect the cartilage and subchondral bone structures of TMJOA by reducing inflammation, cartilage degradation, and chondrocyte apoptosis. In a subsequent clinical study involving 80 patients, compared with an ultrashort wave, ESWT significantly relieved pain in TMD patients and improved temporomandibular joint function indicators and mouth opening restrictions^4^. Although the incidence of cartilage diseases in the knee joint and temporomandibular joint is similar, the field of knee joint orthopedics has more funding and more effective end-stage treatment options^5^.

In conclusion, we recommend ESWT as an adjunctive treatment for TMD in future studies for several reasons: First, there is a lack of effective management strategies for TMD, and new treatments are urgently needed to address this problem. Secondly, there are similarities between knee osteoarthritis and TMD, both in terms of pathogenesis and clinical manifestations. Leveraging the mature experience in treating knee osteoarthritis, introducing ESWT into the treatment of TMD will be highly feasible and cost-effective, providing more options for the currently challenging TMD treatment. Finally, the safety of ESWT applications has been demonstrated in a wide range of clinical practices for different disorders, such as chronic low back pain, erectile dysfunction, and knee osteoarthritis. This suggests that ESWT can be used as a complement to existing therapies and may not impose additional safety burdens on patients. In the future, multidimensional basic research (including the molecular mechanisms of ESWT in anti-inflammatory, analgesic, tissue damage reduction, and tissue repair promotion) and larger-scale, rigorously designed clinical trials will be of great significance in advancing the expansion of ESWT indications and alleviating the pain of TMD patients.

## Ethical approval

Not applicable.

## Consent

Not applicable.

## Source of funding

This research was supported by the Beijing Shunyi District Research and Development Program, Award Number: Shunyi2023Q06.

## Author contribution

W.S. and H.W.: conceptualization; K.H.: funding acquisition. All authors were involved in writing – original draft and writing – review and editing.

## Conflicts of interest disclosure

All authors declare that the research was conducted in the absence of any commercial or financial relationships that could be construed as a potential conflict of interest.

## Research registration unique identifying number (UIN)

Not applicable.

## Guarantor

Wenpeng Song and Hao Wang.

## Data availability statement

The dataset supporting the conclusions of this article is included within the article.

## Provenance and peer review

Not commissioned, externally peer-reviewed.
